# CANNABIDIOL- PROVOKES OR TREATS ANXIETY DISORDERS?

**DOI:** 10.1192/j.eurpsy.2023.1432

**Published:** 2023-07-19

**Authors:** H. Arshad, A. Arshad, M. Khalid, A. R. Khan, F. Arain, S. Khatri

**Affiliations:** 1 Psychiatry, Jinnah Sindh medical university, Karachi; 2 Psychiatry, Allama Iqbal Medical College, Lahore, Pakistan; 3 Psychiatry, Carilion Clinic Virginia Tech, Virginia; 4 Psychiatry, Rutgers New Jersey School of Medicine; 5 Psychiatry, Ocean Medical Center, New Jersey, United States

## Abstract

**Introduction:**

After the introduction of proposal regarding cannabidiol for the treatment of some psychiatric disorders including anxiety, there is confusion if cannabidiol use is associated with the provocation of anxiety symptoms or it can be safely used for the treatment. In nonmedical terms, (Cannabidiol) Cannabis is referred to as Marijuana and has been considered a potential substance of abuse for ages, that raises few questions for its use as a treating agent. It is an interesting area to be explored.

**Objectives:**

Our aim is to find out the implications of Cannabidiol use. We look forward to knowing the mechanism behind cannabidiol being a potential treatment strategy for anxiety.

**Methods:**

A literature search was conducted using the search terms [anxiety] OR [cannabis] OR[ Marijuana] OR [cannabidiol] OR [tetrahydrocannabinol] OR [phytocannabinoids] OR [panic] OR [generalized anxiety] OR [social anxiety] OR [psycholgic distress] OR[psychosis] OR [depression]. The overall search produced 230 results. We included 30 studies relevant to the subject in this review.

**Results:**

Results revealed that anxiety is highly prevalent in individuals with a history of cannabidiol use in comparison to non-users. Symptoms of stress are more pronounced with more frequent cannabidiol use. Chronic users present with more severe symptoms like palpitations and the constant restlessness that are difficult to be managed. The potential role of Cannabinoids in reducing the conditioning of fear can be considered one of the reasons for investigations being done on it. Cannabidiol (Cb1) receptor plays a potential role in producing anxiolytic effects. The side effects of first-line drugs like distorted body shape due to weight gain, sexual health concerns and resistance along with frequent relapses, available for managing anxiety disorders are one of the reasons to consider alternative substances. Though, human testings are still underway, animal models are used currently for experimentation purposes and show positive anxiolytic effects of cannabidiol.

**Conclusions:**

There is increased need to investigate necessary chemical and physiologic changes that are produced within the body in response to cannabidiol use. More investigations should be done on human subjects along with animal studies. Proper guidelines should be shared with practicing physicians so that new and pretested ways are open for the treatment of resistant cases with proper implications of knowledge in clinical settings so that there is minimal chance of abuse of potentially addictive chemicals.

Keywords: Cannabis, Cannabidiol, anxiety, treatment, provocation.

**Disclosure of Interest:**

None Declared

